# Infant regulatory behavior problems during first month of life and neurobehavioral outcomes in early childhood

**DOI:** 10.1007/s00787-018-1243-8

**Published:** 2018-11-03

**Authors:** Elena Toffol, Ville Rantalainen, Marius Lahti-Pulkkinen, Polina Girchenko, Jari Lahti, Soile Tuovinen, Jari Lipsanen, Pia M. Villa, Hannele Laivuori, Esa Hämäläinen, Eero Kajantie, Anu-Katriina Pesonen, Katri Räikkönen

**Affiliations:** 1 Department of Psychology and Logopedics, University of Helsinki, Haartmaninkatu 3, PO Box 21, 00014 Helsinki, Finland; 2 National Institute for Health and Welfare, Helsinki, Finland; 3 University/British Heart Foundation Centre for Cardiovascular Science, Queen’s Medical Research Institute, University of Edinburgh, Edinburgh, UK; 4 Helsinki Collegium for Advance Studies, University of Helsinki, Helsinki, Finland; 5 Department of Obstetrics and Gynaecology, University of Helsinki and Helsinki University Hospital, Helsinki, Finland; 6 Medical and Clinical Genetics, University of Helsinki and Helsinki University Hospital, Helsinki, Finland; 7 Institute for Molecular Medicine Finland/Helsinki Institute of Life Science, University of Helsinki, Helsinki, Finland; 8 Faculty of Medicine and Life Sciences, University of Tampere, Tampere, Finland; 9 Department of Obstetrics and Gynaecology, Tampere University Hospital, Tampere, Finland; 10 HUSLAB and Department of Clinical Chemistry, University of Helsinki and Helsinki University Hospital, Helsinki, Finland; 11 Children’s Hospital, Helsinki University Hospital and University of Helsinki, Helsinki, Finland; 12 PEDEGO Research Unit, MRC Oulu, Oulu University Hospital and University of Oulu, Oulu, Finland

**Keywords:** Regulatory behavior, Infant, Depressive symptoms, Temperament, Neurobehavioral outcomes

## Abstract

Whether infant regulatory behavior problems already in the first month of life indicate an increased risk of childhood neurobehavioral problems, and whether maternal depression in the postpartum and early childhood underpins these associations remain unclear. Altogether, 2049–2364 mothers from the Prediction and Prevention of Pre-eclampsia and Intrauterine Growth Restriction (PREDO) study completed the Neonatal Perception Inventory on regulatory behavior problems at the infant’s age of 15.6 days (SD 3.2, range 1–30), the Infant Behavior Questionnaire-Revised on temperament at 6.5 months (SD 0.9, range 4.2–12.4), and the Ages and Stages Questionnaire-3 on developmental milestones and the Child Behavior Checklist on behavioral problems at 3.5 years (SD 0.7, range 1.9–6.0). Maternal depressive symptoms were measured by the Center for Epidemiological Studies Depression Scale (infancy follow-ups) and Beck Depression Inventory-II (childhood follow-up). Father-rated infant temperament and paternal depressive symptoms were also available (*n* = 1474). Higher levels of infant regulatory behavior problems predicted higher levels of mother- and father-rated negative affectivity temperament (0.13 SD units per SD unit, 95% confidence interval 0.09–0.17; and 0.09, 0.04–0.14, respectively), lower levels of mother-rated orienting/regulation temperament (− 0.09, − 0.13 to − 0.05) and problem-solving skills (− 0.12, − 0.21 to − 0.04), and higher levels of Externalizing (0.07, 0.03–0.11) and Total behavioral problems (0.07, 0.03–0.11). Regulatory behaviors partially mediated the effect of maternal depressive symptoms. Regulatory behavior problems already during the first month of life predict neurobehavioral outcomes, and partially mediate the effect of maternal depressive symptoms. Our study may inform design of interventions aimed at timely prevention in children at risk.

**Electronic supplementary material:**

The online version of this article (10.1007/s00787-018-1243-8) contains supplementary material, which is available to authorized users.

## Introduction

According to Fraley and Roberts [1], constitutional factors operate together with environmental factors and transactional and stochastic processes to create both continuity and change in development. They provide data on the partial continuity of psychological development from the 1st year of life onwards. Multiple studies indeed suggest that certain temperament traits already in the first years of life predict child psychiatric problems and personality traits later in childhood and adulthood [2–4]. Twin studies and genome-wide association studies show the marked, persistent effects of genetics on psychological phenotypes across the life-course [5, 6]. As the genotype stays the same, this contributes to the stability of psychological development, suggesting that early evident behaviors may predict later psychological development.

Research conducted within the developmental origins of health and disease framework in turn highlights the persistent effects that early life, both pre- and postnatal environmental factors have on psychological development, through their effects on organs and cells [7–9]. Life-cycle model of stress further postulates that brain development shows high plasticity in early life, and early life developmental events may influence psychological well-being and mental health through their persistent effects on the developing brain [10]. Attachment theory suggests that mother–child interaction from infancy onwards sets forth long-lasting effects of psychological development [11], and epigenetic studies have shown the persistent effects of pre- and early postnatal developmental factors on gene expression, and of gene expression changes on later psychosocial well-being [12, 13].

Hence, research evidence and theories in developmental psychology both highlight the continuity of psychological development, and the persistent effects of genetics and early-life environmental adversities on psychological development.

Among early-life environmental adversities, special attention is warranted to the role of maternal depression both during pregnancy and in the postpartum period. Indeed, maternal depression is known to be associated with behavioral and emotional, internalizing and externalizing problems as well as impaired cognitive development in the children [14–17], although the exact mechanisms underpinning these associations are not yet clear. While it is plausible that genetic and environmental factors through epigenetic processes contribute to this association, other factors have to be taken into consideration as well. For example, maternal negative affect reduces maternal responsiveness, interferes with a healthy and mature mother–baby interaction, and influences parenting style, which in turn dampen the development of the baby’s regulatory capacities and may eventually lead to cognitive and psychiatric symptoms [18, 19]. Also, it cannot be ignored that maternal negative affect influences maternal perception of child development and behavior, and depressed mothers are more attentive to any emotional or behavioral symptoms, and more likely to perceive their children as difficult, and their development as problematic [14]. While this factor may, therefore, introduce a bias when parental reports are used instead of objective measures to assess child development, the clinical significance of such associations cannot be ignored, because a constant negatively biased perception by a depressed mother can be a risk factor for the future development of her child.

Infant regulatory behavior problems are defined as excessive crying, sleeping and feeding problems, as well as difficulties in self-soothing and mood regulation, that are observable during the first year of life and are a feature of sensory processing disorders and of problems of feeding, sleeping and excessive crying [20]. Around 20% of all infants incur single or multiple regulatory behavior problems [21]. In approximately 90% of them, the problems are, however, transient or intermittent, but a minority have increasing or persistent regulatory problems from early childhood to preschool age [22, 23]. Mounting evidence suggests that infant regulatory behavior problems are early precursors of behavioral and cognitive problems later in childhood. Indeed, individual studies and meta-analyses have shown that crying, feeding and sleeping problems in infancy are associated with temperament traits characterized by high negative affectivity and low activity and sociability at age 3 and 8–10 years [24, 25], as well as increased risk of internalizing, externalizing, conduct and attention-deficit hyperactivity disorder (ADHD) problems at pre-school and school age, and poorer cognitive functioning and academic achievement at age 8–10 years [21, 25–30].

As reported in a recent meta-analysis [21], the majority of the studies to date have focused on regulatory behaviors as assessed after the 3-month age interval that supposedly characterizes the infantile colic period. This has led to the conclusion that persistent regulatory behavior problems, but not necessarily more transient and earlier emerging problems, may index risk of later neurobehavioral problems. Hence, the predictive significance of early regulatory behavior problems, as detected already during the first month of life, is still a matter of debate and has not been explored in detail to date.

It also remains less clear what role maternal depression, which complicates the lives of around 15% of women during the postpartum [31] and increases the risk for subsequent depressive episodes later in life [32, 33], plays in understanding these associations. While maternal depression is well known to be associated with infant regulatory behavior problems and later neurobehavioral outcomes [24, 32, 33], the timing and direction of the effects between maternal depression, infant regulatory behavior problems and childhood neurobehavioral outcomes have not been disentangled yet.

Therefore, the aim of our study was to test if infant regulatory behavior problems as assessed by the mother during the infant’s first 30 days of life are associated with the child’s temperament characteristics at an average age of 6.5 months, and the child’s developmental milestones and psychiatric behavior problems at an average age of 3.5 years as rated by the child’s mother. We also tested associations between infant’s regulatory problems and temperament characteristics at the child’s average age of 6.5 months as rated by the child’s father. A further aim was to examine the interplay between maternal depressive symptoms and these child neurobehavioral outcomes, and also test the role of paternal depression in the analyses of father-rated child temperament. Based on the theoretical frameworks highlighting the continuity of psychological development and also based on previous research findings, we hypothesized that regulatory behavior problems in infancy would be associated with higher scores on infant temperament trait negative affectivity and lower scores on temperament traits extraversion/surgency and orienting/regulation in infancy and lower scores on age-appropriate developmental milestones and higher behavioral problems later in childhood. We also hypothesized that maternal depressive symptoms would be associated with higher infant regulatory behavior problems and with higher scores on infant negative affectivity and lower scores on orienting/regulation, and lower developmental milestones scores and higher behavioral problems later in childhood. In contrast, since previous evidence on the direction of associations between parental depression, infant regulatory behavior problems and later development is scarcer, we performed mediation analyses on the direction of associations more exploratively. We tested these associations in a large prospective cohort of Finnish mothers, fathers and their children, and took into account a number of important covariates.

## Methods

### Participants

Participants come from the Prediction and Prevention of Pre-eclampsia and Intrauterine Growth Restriction (PREDO) study. The cohort comprises 4777 women, recruited in arrival order when attending the first ultrasound screening at 12 + 0–13 + 6 weeks + days of gestation in one of the ten hospital maternity clinics in Southern and Eastern Finland participating in the study, who gave birth to a singleton live child between 2006 and 2010. The cohort profile [34] contains details of the study design and inclusion criteria.

Of these women, 3040 filled-in the regulatory behaviors questionnaire at the infant’s average age of 15.6 days (SD 3.2, range 1–30). Of them, 2364 (77.8%) completed the temperament questionnaire at the child’s average age of 6.5 months (SD 0.9, range 4.2–12.4), 2049 completed the questionnaire on developmental milestones, and 2099 (69.0%) the questionnaire on psychiatric problems at the child’s average age of 3.5 (SD 0.7, range 1.9–6.0) years. Additionally, 1474 fathers filled-in the temperament questionnaire at the child’s 6.5-month follow-up.

Compared to those women who did not complete the infant regulatory behaviors questionnaire within the infant’s first 30 days, women who did had a lower early-pregnancy body mass index (BMI; 24.4 vs. 24.7 kg/m2, SD 4.9 vs. 5.3, *t*(3401.4) = − 2.0, *p *= 0.042), were older (mean age = 31.7 vs. 31.0 years, SD 4.7 vs. 5.2, *t*(3326.8) = 4.4, *p *< 0.001), less likely to smoke throughout pregnancy (3.4% vs. 8.0%, *χ*2= 48.0, *p *< 0.001) and to use alcohol during pregnancy (15.6% vs. 21.5%, *χ*2= 12.0, *p *= 0.001), and more likely to be primiparous (40.5% vs. 35.5%, *χ*2 = 11.3, *p *= 0.001).

The PREDO study protocol was approved by the Ethics Committee of Obstetrics and Gynaecology and Women, Children and Psychiatry of the Helsinki and Uusimaa Hospital District and by the participating hospitals. All participants provided written informed consent. Consent of participating children was provided by parent(s) or guardian(s).

### Infant regulatory behaviors

Infant regulatory behaviors were rated by the mothers using the Neonatal Perception Inventory (NPI) [35]. The NPI consists of 6 questions assessing, on a 5-point Likert scale (no problems to great amount of problems) the expected frequency of infant’s crying, feeding, vomiting (spitting), elimination (bowel movements), sleeping and predictability (adaptation to sleep and eating patterns) behaviors. Mothers were asked to answer the same questions twice: first, they rated the expected behaviors in an “average” and then in the own infant. The final score is computed as a difference score between the own infant and the average infant, with higher scores indicating perceptions of more regulatory behavior problems in own infant. It has been previously reported that the scale yields one factor with an eigenvalue above one which explains 40% of the total variance, and that Cronbach’s alpha of the scale is 0.71 [36]. This lends credence to construct validity and reliability of the scale.

### Infant temperament

Infant temperament was mother- and father-rated with the Infant Behavior Questionnaire-Revised (IBQ-R) [37]. The IBQ-R comprises 191 questions describing the infant’s behavior in different daily situations. Parents are asked to rate how often during the past 2 weeks their infant had behaved or reacted in a way described (1 = never to 7 = always). The IBQ-R yields three main temperament dimensions, negative affectivity, surgency/extraversion, and orienting/regulation. The temperament scales by Rothbart form a golden standard in temperament research due to their good psychometric properties [37–39].

### Child developmental milestones

The Ages and Stages Questionnaires (ASQ) Third edition [40] (translated into Finnish and back-translated and approved by the publisher) is a tool with excellent psychometric properties to screen children requiring further developmental assessment, monitoring or special education [41–43]. It comprises 30 age-appropriate items measuring communication, gross motor, fine motor, problem-solving, and personal and social skills.

Each domain comprises six questions with response ‘yes’ (10) indicating the child can master the skill, ‘sometimes’ (5) if the skill is emerging or occasional, and ‘not yet’ (0) if the child is not able to perform the skill. Scores range from 0 to 60 with the highest value indicating mastering of the skill.

### Child behavioral problems

Child Behavior Checklist for ages 1½–5 (CBCL/1½–5), filled in by the child’s mother, comprises 99 problem items rated on a scale of not true (0) to very true or often true (2), [44]. The CBCL/1½–5 yields scores for three main scales (Internalizing, Externalizing and Total Problems), seven syndrome scales (emotionally reactive, anxious/depressed, somatic complaints, withdrawn, sleep problems, attention problems, and aggressive behavior) and five Diagnostic and Statistical Manual for Mental Disorders-4th Edition (DSM-IV)-oriented scales (affective, anxiety, pervasive developmental, attention deficit/hyperactivity, and oppositional defiant problems). The CBCL/1½–5 has good test–retest reliability, internal consistency and criterion validity [44, 45].

### Maternal and paternal depressive symptoms

At the time of rating the infant’s regulatory behavior problems, the mothers completed the Center for Epidemiological Studies Depression Scale (CES-D) [46], which comprises 20 questions on frequency of depressive symptoms during the past seven days rated on a scale from none of the time (0) to all of the time (3). At the time of rating the infant’s temperament, mothers and fathers completed the CES-D; at the time of rating the child developmental milestones and behavioral problems, the mothers completed the Beck Depression Inventory-II (BDI-II), which comprises 21 questions assessing depressive symptoms during the previous 2 weeks. Each item contains four statements, rated from 0 to 3, reflecting increasing degrees of symptom severity [47].

### Covariates

These included child’s sex (girl or boy), gestational age (weeks) and weight (kg) at birth, maternal age at delivery (years), and early-pregnancy BMI (kg/m2) derived from the Finnish Medical Birth Register (MBR), and child’s age at infant regulatory problems assessment. In addition, we also adjusted for child age at the follow-up in question, either at temperament, behavioral problem, or developmental milestones assessment. Maternal educational attainment (primary/secondary vs. lower tertiary vs. upper tertiary) was self-reported in early pregnancy. While we tested the interplay between maternal and paternal depressive symptoms, infant regulatory behaviors and neurobehavioral outcomes by path analyses (see “Data analyses” below), we also adjusted for their effects in the analyses testing the associations between infant regulatory behaviors and child neurobehavioral outcomes. In additional models, we made further adjustments for parity (primiparous or multiparous) and smoking during pregnancy (no vs. quit during first trimester vs. smoked throughout pregnancy), derived from the MBR, and alcohol use during pregnancy (yes or no), which was self-reported in early pregnancy.

### Data analyses

Associations between infant regulatory behaviors total score and maternal and paternal ratings of temperament, and Total, Internalizing and Externalizing behavioral problem scores were tested using generalized linear models with Gaussian reference distribution. Associations with developmental milestones total score and behavioral problems syndrome- and DSM-IV-scores were tested using Tobit regression due to the variable distributions. Developmental milestones total score was highly skewed with a ceiling effect: upper limit of the scale score is 60 indicating that the child masters the age-specific skill, but the scale does not distinguish performance of children above this highest value. Behavioral problems syndrome- and DSM-IV-scores were truncated at 50 leading to a skewed distribution with a floor effect. Further, associations with the developmental milestones domain scores were tested with ordered logistic regression as the domain scores have a 12-point ordinal scale. We made adjustments for covariates as described in detail in the “Results” section and footnotes of Tables. These analyses were conducted using IBM SPSS statistics software version 24.0 (IBM Corp., Armonk, NY) and the R program [48].

To study the interplay between maternal and paternal depressive symptoms, infant regulatory behavior problems and child neurobehavioral outcomes, we used path analysis using the IBM SPSS Amos software version 24.0 with 5000 bootstrapped samples.

To facilitate interpretation, continuous outcome variables (with the exception of outcome variables in Tobit and ordered logistic regression), predictors and covariates were standardized to the mean of 0 and SD of 1. In all the analyses, two-tailed *p* values of < 0.05 were considered significant.

## Results

Characteristics of the study participants are reported in Table 1. Both women with primary or secondary education and women with lower tertiary education reported higher regulatory behavior problems in their infants than women with upper tertiary education [mean differences in SD units 0.12, and 0.10, *F*(3034) = 4.07, *p *= 0.018, respectively]. Higher infant behavior problem scores were also associated with higher maternal early-pregnancy BMI (*r* = 0.04, *p *= 0.045), infant’s lower birth weight (*r *= − 0.04, *p* = 0.018), and higher maternal depressive symptoms at rating the infant’s regulatory behaviors (*r *= 0.22, *p *< 0.001). Infant’s gestational age at birth, preterm birth, sex, maternal age at delivery and paternal depression at the time of rating the infant’s temperament were not significantly associated with infant’s regulatory behaviors. When stratifying by child’s sex, higher regulatory behavior problem scores were associated with lower gestational age in girls (*r *= − 0.05, *p *= 0.042), but not in boys (*p *= 0.826). No other sex differences in these associations were found.

**Table 1:** Characteristics of the study participants

	Mean (SD)/*n* (%)
Pregnancy and birth (*n* = 3040)	
Maternal characteristics	
Educational attainment	
Primary or secondary	1242 (40.9)
Lower tertiary	783 (25.8)
Upper tertiary	1011 (33.3)
Age at delivery (years)	31.7 (4.7)
Early-pregnancy body mass index (kg/m2)	24.4 (4.9)
Primiparous	1217 (40.5%)
Alcohol use during pregnancy	469 (15.6%)
Smoking during pregnancy	
No	2832 (93.2%)
Quit during first trimester	102 (3.4%)
Smoking throughout pregnancy	104 (3.4%)
Child characteristics	
Sex (boys)	1554 (51.5%)
Birth weight (g)	3536 (510)
Gestational age (weeks)	39.9 (1.5)
Preterm birth (< 37 gestational weeks)	107 (3.5%)
Follow-up at 15.6 days (*n* = 3040)	
Maternal characteristics	
Center for Epidemiological Studies Depression Scale sum score (*n* = 2992)	10.7 (7.9)
Child characteristics	
Age (days)	15.6 (3.2)
Neonatal Perception Inventory score	− 1.3 (2.6)
Follow-up at 6.5 months (*n* = 2364)	
Maternal characteristics	
Center for Epidemiological Studies Depression Scale sum score (*n* = 2343)	9.3 (7.2)
Paternal characteristics	
Center for Epidemiological Studies Depression Scale sum score (*n* = 1451)	7.5 (6.0)
Child characteristics	
Age (months)	6.5 (0.9)
Temperament	
Mother-rated (*n* = 2364)	
Surgency/extraversion	4.6 (0.6)
Negative affectivity	2.9 (0.6)
Orienting/regulation	4.9 (0.6)
Father-rated (*n* = 1474)	
Surgency/extraversion	4.5 (0.6)
Negative affectivity	3.0 (0.6)
Orienting/regulation	4.7 (0.6)
Follow-up at 3.5 years (*n* = 2049–2099)	
Maternal characteristics	
Beck Depression Inventory sum score	6.5 (6.4)
Child characteristics	
Age (years)	3.5 (0.7)
Behavior problems (*n* = 2099)	
Internalizing	45.8 (9.5)
Externalizing	47.4 (9.1)
Total	46.3 (9.2)
Developmental milestones (*n* = 2049)	
Ages and Stages Questionnaire-3 total mean	53.9 (6.2)

### Infant regulatory behavior problems and temperament at 6.5 months

Table 2 shows that higher levels of infant regulatory behavior problems were associated with higher levels of mother-rated infant negative affectivity and lower levels of orienting/regulation across all adjustment models. Table 2 shows that higher levels of infant regulatory behavior problems were also associated with higher levels of father-rated infant negative affectivity across all adjustment models. Mother- and father-rated infant temperament was correlated as follows: negative affectivity, *r* = 0.52, *p *< 0.001; surgency/extraversion, *r* = 0.33, *p *< 0.001; orienting/regulation, *r* = 0.35, *p *< 0.001.

**Table 2:** Associationsa between infant’s regulatory behavior problems during the first 30 days of life, temperament at the age of 6.5 months, and developmental milestones and psychiatric problems at the age of 3.5 years

	Model 1a			Model 2b			Model 3c			Model 4d		
	*B* (SE)	95% CI	*p*	*B* (SE)	95% CI	*p*	*B* (SE)	95% CI	*p*	*B* (SE)	95% CI	*p*
Temperament												
Surgency/extraversion												
Mother-rated	0.00 (0.02)	[− 0.04, 0.04]	0.953	0.00 (0.02)	[− 0.04, 0.04]	0.833	− 0.01 (0.02)	[− 0.05, 0.03]	0.745	0.02 (0.02)	[− 0.02, 0.06]	0.388
Father-rated	0.00 (0.03)	[− 0.05, 0.05]	0.972	0.00 (0.03)	[− 0.05, 0.05]	0.948	− 0.01 (0.03)	[− 0.06, 0.05]	0.830	0.01 (0.03)	[− 0.05, 0.06]	0.814e
Negative affectivity												
Mother-rated	*0.18* (*0.02*)	[0*.14, 0.22*]	< *0.001*	*0.18* (*0.02*)	[*0.14, 0.22*]	< *0.001*	*0.18* (*0.02*)	[*0.14, 0.22*]	< *0.001*	*0.13* (*0.02*)	[*0.09, 0.17*]	< *0.001*
Father-rated	*0.11* (*0.03*)	[0*.06, 0.16*]	< *0.001*	*0.11* (*0.03*)	[*0.06, 0.16*]	< *0.001*	*0.11* (*0.03*)	[*0.05, 0.16*]	< *0.001*	*0.09* (*0.03*)	[*0.04, 0.14*]	*0.001*d
Orienting/regulation												
Mother-rated	− *0.12* (*0.02*)	[− *0.16,* − *0.08*]	< *0.001*	− *0.12* (*0.02*)	[− *0.16,* − *0.08*]	< *0.001*	− *0.13* (*0.02*)	[− *0.17*, − *0.09*]	< *0.001*	− *0.09* (*0.02*)	[− *0.13,* − *0.05*]	< *0.001*
Father-rated	− 0.02 (0.03)	[− 0.07, 0.03]	0.476	− 0.02 (0.03)	[− 0.07, 0.03]	0.451	− 0.03 (0.03)	[− 0.08, 0.03]	0.333	− 0.01 (0.03)	[− 0.06, 0.05]	0.767e
Behavior problems												
Internalizing	*0.11* (*0.02*)	[*0.07, 0.15*]	< *0.001*	*0.10* (*0.02*)	[*0.06, 0.15*]	< *0.001*	*0.10* (*0.02*)	[*0.06, 0.14*]	< *0.001*	0.03 (0.02)	[− 0.01, 0.07]	0.152
Externalizing	*0.14* (*0.02*)	[*0.09, 0.18*]	< *0.001*	*0.13* (*0.02*)	[*0.09, 0.17*]	< *0.001*	*0.12* (*0.02*)	[*0.08, 0.17*]	< *0.001*	*0.07* (*0.02*)	[*0.03, 0.11*]	*0.001*
Total	*0.14* (*0.02*)	[*0.10, 0.19*]	< *0.001*	*0.14* (*0.02*)	[*0.10, 0.18*]	< *0.001*	*0.13* (*0.02*)	[*0.09, 0.17*]	< *0.001*	*0.07* (*0.02*)	[*0.03, 0.11*]	*0.001*
Developmental milestones												
Total score	− *0.35* (*0.15*)	[− *0.63,* − *0.06*]	*0.018*	− *0.33* (*0.15*)	[− *0.62,* − *0.05*]	*0.023*	− 0.28 (0.15)	[− 0.56, 0.00]	0.053	− 0.04 (0.15)	[− 0.34, 0.26]	0.806

B refers to unstandardized beta coefficient and indicates one SD unit change in outcome per one SD unit change in infant regulatory behaviors. B for the Developmental milestones Total score indicates one unit change per one SD unit change in infant regulatory behaviors. 95% CI refers to 95% confidence interval. Significant associations are marked in italics

aModel 1: adjusted for child’s sex and ages when filling-in the infant regulatory behavior problems and the follow-up questionnaires

bModel 2: adjusted for Model 1 + birth weight and gestational age

cModel 3: adjusted for Model 2 + maternal age, early-pregnancy body mass index and education

dModel 4: Model 3 + maternal depression at filling-in the questionnaires

eModel 4 in fathers: Model 3 + maternal depression at rating the infant’s regulatory behavior problems and paternal depression at rating the infant’s temperament

### Infant regulatory behavior problems and developmental milestones at 3.5 years

**Fig. 1:**
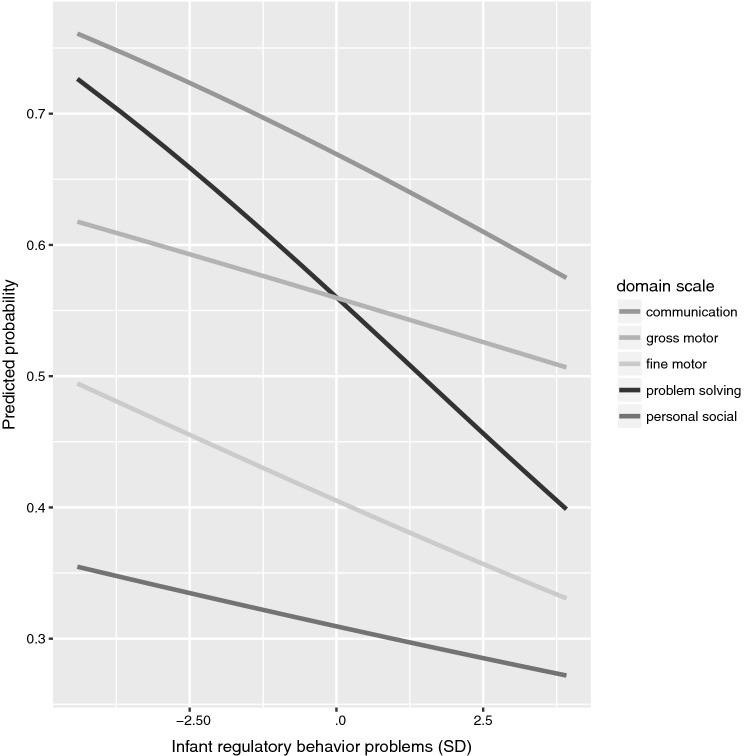
Predicted probabilities of scoring 60 on the developmental milestone domain scales by infant regulatory behavior problems

Higher levels of infant regulatory behavior problems were associated with a lower total score on the developmental milestones (*p *= 0.018) (Table 2). This association did not survive adjustment for maternal perinatal characteristics or depressive symptoms (Table 2). Analyses of the domains of developmental milestones showed that higher levels of infant regulatory behavior problems were associated with a lower score on problem-solving skills (Online Resource 1). Figure 1 displays this association, which remained significant across all adjustment models, and shows that the predicted probability of scoring higher on problem-solving skills decreased by increasing levels of regulatory behavior problems during the first month of life.

### Infant regulatory behavior problems and behavioral problems at 3.5 years

Higher levels of infant regulatory behavior challenges were significantly associated with higher levels of Total, Internalizing and Externalizing behavioral problems in early childhood (Table 2). Associations with Total and Externalizing problems remained significant across all adjustment models; the association with Internalizing problems did not survive adjustment for maternal depressive symptoms.

The Table in the Online Resource 2 shows associations with the syndrome- and DSM-IV-oriented behavioral problems. Except for the DSM-IV Pervasive Developmental Problems scale, all the associations were significant and showed more problems in infants with higher levels of regulatory behavior challenges.

Sensitivity analyses conducted by excluding 107 children born preterm did not change any of the results reported above. The results did not change when we made further adjustments for parity, maternal smoking and alcohol use during pregnancy (data not shown).

### Interplay between maternal and paternal depressive symptoms, infant regulatory behavior problems, and early childhood neurobehavioral outcomes

Infant regulatory behavior problems were not associated with mother- or father-rated surgency/extraversion, or with father-rated orienting/regulation temperament traits. Moreover, the associations with mother-rated developmental milestones score did not survive adjustment for maternal perinatal characteristics. Therefore, we did not pursue testing the interplay between maternal or paternal depressive symptoms, infant regulatory behavior problems and these offspring neurobehavioral outcomes.

**Fig. 2:**
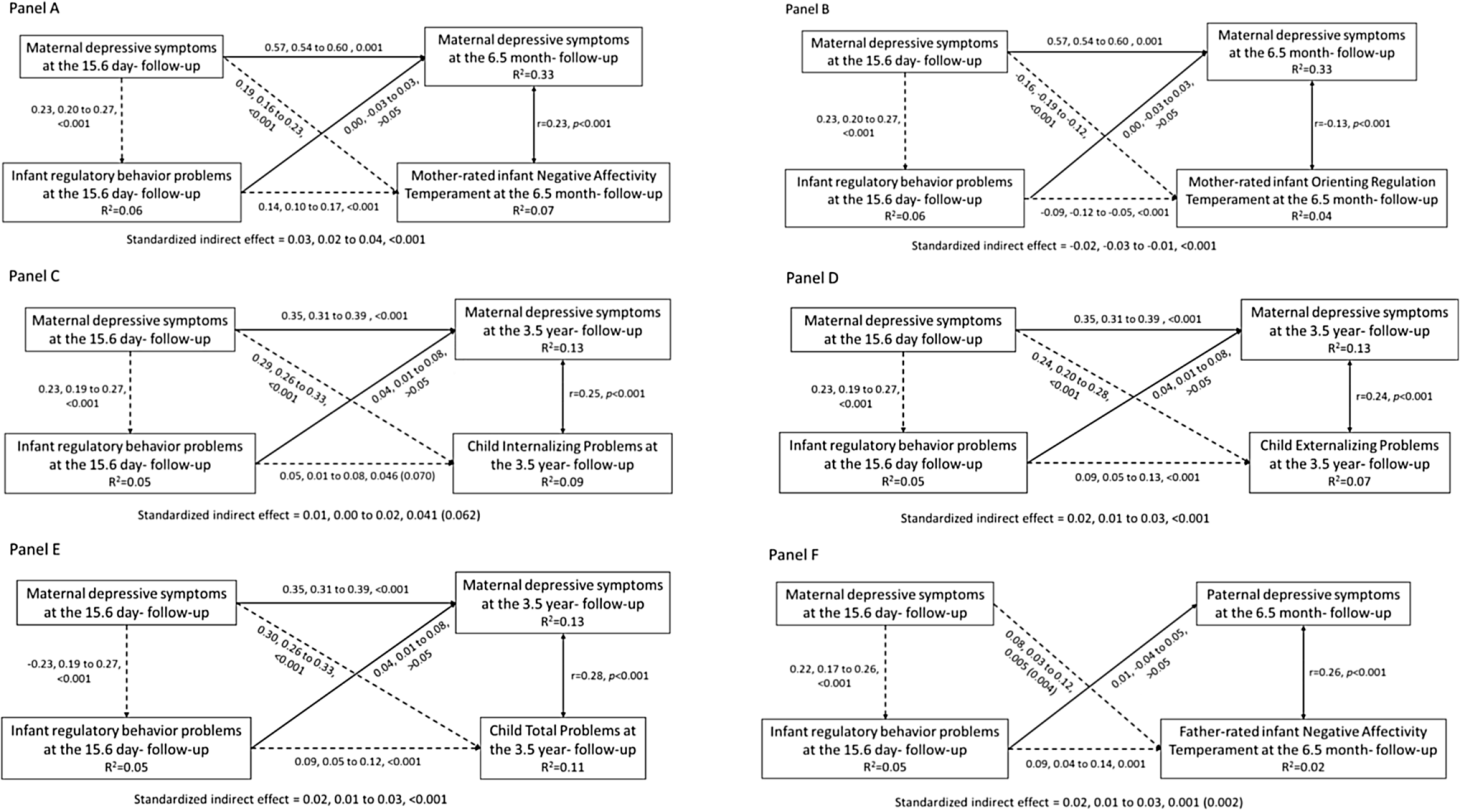
Mediation analyses show the associations between maternal depressive symptoms at rating the infant’s regulatory behaviors, maternal or paternal depression at follow-ups, maternal ratings of infant regulatory behavior problems, and maternal ratings of infant’s negative affectivity (**a**), orienting/regulation (**b**), total (**c**), externalizing (**d**) and internalizing (**e**) behavioral problems, and paternal ratings of negative affectivity (**f**). Dashed lines represent the direct and indirect effects of maternal depression at 15.6 days on later child outcome via infant regulatory behavior problems. Solid lines represent direct effects; double arrowed lines represent correlations. Numbers represent unadjusted unstandardized coefficients, 95% confidence intervals, and *p* values; number in parentheses represent *p* values adjusted for child’s sex, age, birth weight, gestational age, and maternal age, early-pregnancy body mass index and education, when different from unadjusted *p* values. *R*2 represents the proportion of variance accounted for by maternal depressive symptoms and infant regulatory behavior problems

Figure 2 shows that maternal depressive symptoms when rating the infant’s regulatory behaviors, were associated with 15.6-day-old infant’s regulatory behavior problems. Maternal depressive symptoms also had direct effects on 6.5-month-old infant’s negative affectivity (panel a) and orienting/regulation temperament traits (panel b), and on 3.5-year-old child’s total (panel c), externalizing (panel d) and internalizing (panel e) behavioral problems. Maternal depressive symptoms also had indirect effects on these neurobehavioral outcomes via infant’s regulatory behavior problems. Maternal depressive symptoms and infant regulatory behavior problems accounted for 2–11% of the total variance of negative affectivity, orienting/regulation, and total, externalizing and internalizing problems. Figure 2 also shows that infant’s regulatory behavior problems did not have an effect on maternal depressive symptoms at the time of rating the child’s neurobehavioral outcomes, in models accounting for maternal depressive symptoms when rating the infant’s regulatory behavior problems. A path model including paternal depressive symptoms at the time of rating the infant’s negative affectivity did not change the direct and indirect effects of maternal depressive symptoms on child neurobehavioral outcomes, and infant’s regulatory behaviors did not predict paternal depressive symptoms (Fig. 2, panel f).

## Discussion

The main findings of this study are that regulatory behavior problems perceived by the mother in her less than 1-month-old infant predicted more difficult temperamental traits when the infant was aged 6.5 months, and higher levels of behavioral problems and lower scores on developmental milestones when the child was aged 3.5 years. More specifically, higher levels of regulatory behavior problems in own in comparison to an average infant were associated with higher levels of infant negative affectivity temperament in both maternal and paternal ratings, and lower levels of orienting/regulation temperament in maternal ratings. Perceptions of higher regulatory behavior problems in own in comparison to an average infant were also associated with higher levels of child Externalizing and Total behavioral problems and lower problem-solving skills in the developmental milestones measure in maternal ratings. These associations were not explained by a number of important covariates including maternal early-pregnancy BMI, age at delivery, educational attainment, parity, smoking and alcohol use during pregnancy, and child’s sex, age at testing, gestational age and birthweight. Excluding children born preterm did not change any of the findings. Our findings, thus, provide novel information that already during the first 30 days of life, maternal perceptions of infant regulatory behavior problems have predictive validity for offspring’s later neurobehavioral outcomes. Our finding that regulatory behavior problems not only predicted child’s mother-rated negative affectivity temperament, but also predicted father-rated negative affectivity temperament is in support of this idea. In agreement with earlier studies that have, with only few exceptions [25, 26, 30], used exclusively mother ratings as the primary informant source for child neurobehavioral outcomes [23, 24, 27–29], these findings, thus, suggest that early-emerging regulatory behavior problems are predictive of child’s later neurobehavioral outcomes. Our findings, however, suggest that this may not only hold true for problems that are more persistent and still emergent after the typical infantile 3-month colic period, but also for those problems that emerge earlier. These findings may have long-term clinical significance as parent ratings of childhood conduct, hyperkinetic and emotional problems have been shown to be predictive of any mental, anxiety, mood, antisocial personality, substance use and/or psychotic disorder diagnosis in adulthood in boys [49], and childhood subthreshold psychiatric behavioral problems have been shown to predict adverse adult outcomes in related to health, the legal system, personal finances, and social functioning in both girls and boys [50].

While maternal postpartum depression increases the mother’s risk for later depressive episodes, and also increases the risk of infant regulatory behavior problems and neurobehavioral adversities, our study showed that the associations between infant regulatory behavior problems and childhood neurobehavioral outcomes were not explained by maternal depressive symptoms in the early childhood period. In the analyses of child’s father-rated negative affectivity temperament, father’s depressive symptoms did not account for its association with infant regulatory behaviors. Yet, our results from path analyses revealed that maternal depressive symptoms in the infancy stage had a direct effect on the child’s neurobehavioral outcomes. The path analyses also showed that partially the effect of maternal depressive symptoms in the infancy stage was mediated through maternal ratings of the infant’s regulatory behavior problems. Infant regulatory behavior problems did not, however, contribute to increase the highly stable maternal depressive symptoms from infancy to childhood; in the childhood stage, maternal depressive symptoms were highly correlated with childhood neurobehavioral outcomes, showing that cross-sectionally they are highly related. Although previous studies have also shown associations between maternal depression in infancy and child neurobehavioral outcomes [16, 33], these findings of mediation effects through regulatory behavior problems in early infancy are novel and offer insight into the early environmental and intrinsic mechanisms that may underpin the adverse neurobehavioral development. In terms of clinical implications, these observations emphasize the importance of identifying women suffering from depressive symptoms as early as possible after childbirth or, given that postpartum depression commonly has its onset already during pregnancy [51], even before childbirth. This identification will benefit not only the well-being of the mother, but also the well-being of the child, and may reduce the risk for future neurobehavioral adversities.

The study of early regulatory behavior problems is complicated and limited by a lack of clear diagnostic criteria in the definition of infant regulatory behavior problems [21] and, thus, of a gold-standard assessment tool. The majority of the studies have used different, mostly non-validated assessment tools, including parent interviews, questionnaires, diaries or observations [21]. Even though Santos et al. [28] have used the mother’s perception of their infants relative to same-age average infants to define “crying babies”, to the best of our knowledge, this is the first study to use the NPI to specifically target regulatory problems in newborns and their associations with the child neurobehavioral development. By assessing regulatory problems with the NPI, we were able to replicate previous findings obtained via other instruments. Our findings, thus, provide support for the use of the NPI as a valid, easily administrable questionnaire to screen for regulatory behavior problems in newborns. The NPI may have clinical utility as it may provide important information for health-care providers and guide for targeted and timely preventive interventions in those at risk.

### Strengths and limitations

Strengths of our study include a large sample size and the prospective design with follow-ups at different developmental stages, and the use of multiple instruments to assess children’s neurobehavioral outcomes from multiple perspectives. A further strength is that we used maternal and paternal ratings, though only for child temperament. Furthermore, while most of the studies to date have been conducted in selected samples of clinically referred children, or children born with various risks, we were able to show the predictive role of early regulatory behaviors in a large sample of children who varied in these risks. We were also able to account for multiple of these risk factors in our analyses, and our findings also held when we excluded children born preterm.

This study has a number of limitations. First, infant regulatory behavior problems, temperament, and early childhood developmental milestones and behavioral problems were all mother- (or father-) rated. This is also the case of most of the previous studies [23, 24, 27, 29], with only one additionally having fathers and teachers as other informants [25], one measuring child intelligence quotient with neuropsychological tests and attention-deficit/hyperactivity disorder with a structured parent interview [26], and one having cognitive assessments carried out by trained pediatricians [30]. In a large prospective epidemiological study like this, objective clinical assessments by experts are, however, less feasible, and the validated and reliable measurement tools that we used provided us a sensible option. Moreover, infant regulatory behavior problems were maternal expectations in comparison to an average infant. While this method aims at reducing the potential bias that may result in too positive or negative maternal perceptions of infant behaviors, it may still carry a bias that is inherent in all parental reports. Our findings, however, correspond with previous literature, and we were able to replicate the findings of infant regulatory behavior problems and negative affectivity temperament in father ratings. This suggests that the NPI is able to capture the mother–infant dyads at risk for future adverse child outcomes. Yet, our findings cannot be generalized to diagnosed problems, disorders and developmental delay. Furthermore, behavioral problems and neurodevelopmental milestones in early childhood were only mother rated, and further studies are needed to see whether similar findings would be obtained with father- or teacher-rated questionnaires on these outcomes, with mental disorder diagnoses extracted from psychiatric interviews or with neuropsychological test scores on the development of motor, communication, social or problem-solving skills. Although temperament traits were rated by both parents, future research on them may also benefit from observational ratings. Furthermore, the infants were very young at the age of NPI assessment with yet limited time spent with their mothers, and there was noticeable variation in infant age at NPI assessment. Although all analyses were adjusted for age at NPI assessment, future research on infant regulatory problems could benefit from assessment during a specific and narrower time frame, for example 4–6 weeks postpartum, when most mother–infant dyads are somewhat more adjusted to the new context. Our study findings cannot be generalized to samples that differ in characteristics from our study sample. Also, loss to follow-up has to be kept in mind in interpreting the study findings. We do not know what the underlying mechanisms explaining the associations are, above and beyond maternal and paternal depressive symptoms, but they may include compromised attachment bond and lower rates of breastfeeding, which are associated with maternal depression. Epigenetic embedding and genetic vulnerability factors may also underlie.

## Conclusions

Our findings demonstrate that maternal perceptions of infant regulatory behavior problems already during the first 30 days of life predict neurobehavioral outcomes in infancy and early childhood in mother ratings, and also predict infant’s negative affectivity temperament in father ratings. Maternal depressive symptoms were associated with infant regulatory behaviors, and infant regulatory behavior problems partially mediated the effect of maternal depressive symptoms in infancy on the child’s neurobehavioral outcomes. Infant regulatory behavior problems did not, however, contribute to increasing maternal depressive symptoms or predict paternal symptomatology over the follow-up. These findings, thus, suggest that the screening for early regulatory behavior problems via the NPI may allow early identification of children at risk for neurobehavioral adversities, and that identification and treatment of maternal depression during the first postpartum months may contribute to preventing long-term adverse outcomes in the offspring.

## Electronic supplementary material

Below is the link to the electronic supplementary material.

Supplementary material 1 (PDF 327 kb)
