# Supplementary material for: Infant regulatory behavior problems during first month of life and neurobehavioral outcomes in early childhood
Source: Eur Child Adolesc Psychiatry. 2018 Nov 3;28(6):847–59. doi: 10.1007/s00787-018-1243-8 (PMC6555774; doi:10.1007/s00787-018-1243-8)
Supplement: Supplementary file 1 — Supplementary material 1 (PDF 327 kb) [file 787_2018_1243_MOESM1_ESM.pdf]

European Child and Adolescent Psychiatry

Infant regulatory behavior problems during first month of life and neurobehavioral outcomes in early childhood

Elena Toffol, Ville Rantalainen, Marius Lahti-Pulkkinen, Polina Girchenko, Jari Lahti, Soile Tuovinen, Jari Lipsanen, Pia M Villa, Hannele Laivuori, Esa Hämäläinen, Eero Kajantie, Anu-Katriina Pesonen, Katri Räikkönen

Correspondence concerning this article should be addressed to Marius Lahti-Pulkkinen, Department of Psychology and Logopedics, PO Box 21 (Haartmaninkatu 3), FI-00014 University of Helsinki, Finland. Email: marius.lahti-pulkkinen@helsinki.fi.

Supplementary tables

Online Resource 1.

Associations between infant's regulatory behavior problems during the first 30 days of life and domains of developmental milestones at the age of 3.5 years

	Model 1[a]			Model 2[b]			Model 3[c]			Model 4[d]		
Domains of developmental milestones	*B (SE)*	*95% CI*	*p*	*B (SE)*	*95% CI*	*p*	*B (SE)*	*95% CI*	*p*	*B (SE)*	*95% CI*	*p*
Communication	*-0.10 (0.05)*	[*-0.19, -0.01*]	*.026*	*-0.10* (0.05)	[*-0.19, -0.01*]	*.026*	-0.08 (0.05)	[-0.17, 0.01]	.076	-0.04 (0.05)	[-0.14, 0.05]	.371
Gross motor	-0.06 (0.04)	[-0.15, 0.02]	.150	-0.06 (0.04)	[-0.14, 0.03]	.167	-0.05 (0.04)	[-0.14, 0.03]	.207	0.00 (0.05)	[-0.09, 0.09]	.963
Fine motor	-0.08 (0.04)	[-0.16, 0.00]	.059	-0.07 (0.04)	[-0.15, 0.01]	.076	-0.06 (0.04)	[-0.14, 0.02]	.137	-0.01 (0.04)	[-0.09, 0.07]	.810
Problem solving	*-0.17 (0.04)*	[*-0.26, -0.09*]	*<.001*	*-0.17 (0.04)*	[*-0.25, -0.08*]	*<.001*	*-0.17 (0.04)*	[*-0.25, -0.08*]	*<.001*	*-0.12 (0.05)*	[*-0.21, -0.04*]	*.006*
Personal social	-0.05 (0.04)	[-0.12, 0.03]	.255	-0.04 (0.04)	[-0.12, 0.04]	.283	-0.04 (0.04)	[-0.12, 0.04]	.341	0.02 (0.04)	[-0.07, 0.10]	.683

Note. B refers to unstandardized beta coefficient and indicates one step change in outcome per one SD unit change in infant regulatory behaviors. 95% CI refers to 95% Confidence Interval. Significant associations across models 1-4 are marked in italics.
[a] Model 1: adjusted for child's sex and age when filling-in the questionnaires. [b] Model 2: adjusted for Model 1 + birth weight and gestational age. [c] Model 3: adjusted for Model 2 + maternal age, early-pregnancy body mass index and education. [d] Model 4: Model 3 + maternal depression at filling-in the questionnaires.

Online Resource 2.

Associations between infant's regulatory behavior problems during the first 30 days of life and syndrome- and DSM-IV- oriented behavioral problems at the age of 3.5 years

	Model 1[a]			Model 2[b]			Model 3[c]			Model 4[d]		
Behavioral problems	*B (SE)*	*95% CI*	*p*	*B (SE)*	*95% CI*	*p*	*B (SE)*	*95% CI*	*p*	*B (SE)*	*95% CI*	*p*
Syndrome scales												
Emotionally reactive	*0.79 (0.20)*	*[0.41, 1.18]*	*<.001*	*0.78 (0.20)*	*[0.40, 1.16]*	*<.001*	*0.72 (0.19)*	*[0.34, 1.10]*	*<.001*	0.28 (0.19)	[-0.10, 0.66]	.150
Anxious/Depressed	*0.61 (0.17)*	*[0.27, 0.95]*	*.001*	*0.60 (0.17)*	*[0.26, 0.94]*	*.001*	*0.57 (0.17)*	*[0.23, 0.91]*	*.001*	0.20 (0.18)	[-0.15, 0.54]	.260
Somatic complaints	*0.90 (0.26)*	*[0.40, 1.40]*	*<.001*	*0.88 (0.26)*	*[0.38, 1.38]*	*.001*	*0.81 (0.26)*	*[0.31, 1.31]*	*.002*	0.30 (0.26)	[-0.21, 0.80]	.246
Withdrawn	*0.47 (0.15)*	*[0.17, 0.76]*	*.002*	*0.45 (0.15)*	*[0.15, 0.74]*	*.003*	*0.42 (0.15)*	*[0.13, 0.72]*	*.005*	0.03 (0.15)	[-0.27, 0.32]	.855
Sleep problems	*0.99 (0.17)*	*[0.65, 1.32]*	*<.001*	*0.97 (0.17)*	*[0.64, 1.31]*	*<.001*	*0.96 (0.17)*	*[0.62, 1.29]*	*<.001*	*0.61 (0.17)*	*[0.27, 0.95]*	*<.001*
Attention problems	*0.63 (0.14)*	*[0.36, 0.90]*	*<.001*	*0.61 (0.14)*	*[0.34, 0.88]*	*<.001*	*0.55 (0.14)*	*[0.28, 0.81]*	*<.001*	*0.30 (0.14)*	*[0.03, 0.56]*	*.032*
Aggressive behaviour	*1.10 (0.21)*	*[0.68, 1.51]*	*<.001*	*1.09 (0.21)*	*[0.68, 1.51]*	*<.001*	*1.02 (0.21)*	*[0.61, 1.43]*	*<.001*	*0.60 (0.21)*	*[0.19, 1.01]*	*.005*
DSM-IV-oriented scales												
Affective Disorders	*0.60 (0.13)*	*[0.34, 0.86]*	*<.001*	*0.59 (0.13)*	*[0.33, 0.85]*	*<.001*	*0.56 (0.13)*	*[0.30, 0.82]*	*<.001*	*0.29 (0.13)*	*[0.03, 0.55]*	*.030*

Anxiety Disorders	*0.95 (0.21)*	*[0.54, 1.35]*	*<.001*	*0.94 (0.21)*	*[0.54, 1.34]*	*<.001*	*0.89 (0.21)*	*[0.49, 1.30]*	*<.001*	0.39 (0.21)	[-0.01, 0.80]	.057
Pervasive Developmental problems	*0.46 (0.18)*	*[0.10, 0.81]*	*.012*	*0.44 (0.18)*	*[0.08, 0.79]*	*.015*	*0.39 (0.18)*	*[0.04, 0.74]*	*.031*	-0.03 (0.18)	[-0.39, 0.33]	.877
ADHD problems	*0.77 (0.16)*	*[0.47, 1.08]*	*<.001*	*0.76 (0.16)*	*[0.45, 1.07]*	*<.001*	*0.70 (0.16)*	*[0.39, 1.01]*	*<.001*	*0.40 (0.16)*	*[0.09, 0.71]*	*.012*
Oppositional defiant problems	*0.77 (0.17)*	*[0.43, 1.11]*	*<.001*	*0.76 (0.17)*	*[0.42, 1.10]*	*<.001*	*0.70 (0.17)*	*[0.36, 1.04]*	*<.001*	*0.38 (0.18)*	*[0.04, 0.73]*	*.029*

Note. B refers to unstandardized beta coefficient and indicates one unit change in outcome per one SD unit change in infant regulatory behaviors. 95% CI refers to 95% Confidence Interval; ADHD refers to Attention Deficit Hyperactivity Disorder. Significant associations across models 1-4 are marked in italics.

[a] Model 1: adjusted for child's sex and age when filling-in the questionnaires. [b] Model 2: adjusted for Model 1 + birth weight and gestational age. [c] Model 3: adjusted for Model 2 + maternal age, early-pregnancy body mass index and education. [d] Model 4: Model 3 + maternal depression at filling-in the questionnaires.
